# Collectanea Medica: Consisting of Anecdotes, Facts, Extracts, Illustrations, &c.

**Published:** 1825-10

**Authors:** 


### COLLECTANEA MEDICA:

CONSISTING OF

### ANECDOTES, FACTS, EXTRACTS, ILLUSTRATIONS, &c.

***Relating to the History or the Art of Medicine, and the Collateral Sciences.***

---

Floriferus, ut apes, in saltibus omnia libant,
Omnia nos, itidem, depascimur aurea dicta.

---

ART. I.—*On the Use of Iodine in Bronchocele; being the Result of* Dr. MANSON'S *Experience in One Hundred and Twenty Cases.*—[Extracted from "*Medical Researches on the Effects of Iodine, in Bronchocele, Paralysis, Chorea, Scrofula, Fistula Lachrymalis, Deafness, Dysphagia, White Swelling, and Distortions of the Spine.* By ALEXANDER MANSON, M.D. Physician to the General Hospital, and St. Mary's Hospital and Dispensary, Nottingham."—8vo. pp. 452. Longman, London.]

FIFTEEN cases in detail precede the table; and, of these, twelve were "cured,"—one "almost cured,"—one was "recovering,"—and in the other the result was not known.

***A Tabular View of the other Cases of Bronchocele, treated by the exhibition of Iodine, in my Hospital and Private Practice, between the 24th of March, 1821, and the 23d of March, 1824.***

*Abbreviatians employed in this Table:*—P.P. for Private Patient; I.P.H. for In-Patient of the Hospital; O.P.H. for Out-Patient of the Hospital. In the column for the Disease, the Figures with y. imply the number of Years, and with m. the number of Months, that the disease has been present. L. prefixed to Bronchocele, signifies that it was larger than usual; and F. M. B. S. A. and C. following the disease, that the Father, Mother, Brother, Sister, Aunt, or Cousin, laboured under Bronchocele; p. and m. prefixed to any of these capitals, signify Paternal and Maternal.

| *No.* | *Name and Age.* | *Residence.* | *Admitted,* 1821. | *Disease, &c.* | *Treatment, &c.* | *When and how Discharged.* |
|---|---|---|---|---|---|---|
| 16 | Miss J. N. æt. 13, P.P. .. | Nottingham | March 24th | Bronchocele | Tæ. Iodini gutt. x. ad xv. ter in die. Pæ. Aloes C. p. r. n. | July 7, 1821—Cured |
| 17 | William Holmes, 10, P.P... | Nottingham | April 10th | Bronchocele | Pulv. Aper. p. r. n. et Tæ. Iodini gutt. x. ter in die ex aquæ ℥ ss. | July 1821—Discharged, cured |
| 18 | Sarah Carlin, 17, I.P.H. .. | Eastwood | April 17th | Bronchocele, &c. | Mist. Purg. ℥ iss. p. r. n. et Tæ. Iodini gutt. xv. ad xx. ter in die ex aquæ ℥ i. | May 12, 1821—Cured. |
| 19 | Eliza Cliff, 18, P.P. ...... | Arnold | April 23d | Bronchocele, Dyspnœa, &c. 4 y. | Spongia ust.; T. Iodini gutt. xv. ad xx. ter in die. | Relieved; not seen since May 12, 1821. |
| 20 | F. Huntington, 18, O.P.H. | Nottingham | May 8th | Bronchocele, &c. | T. Iodini gutt. x. ad gutt. xxx. ter in die. | Dec. 28, 1821—Cured. |
| 21 | C. Wylde, 31, O.P.H....... | Nottingham | May 8th | Bronchocele, &c. | Mist. Purg. p. r. n.; T. Iodini gutt. xv. ad xxxv. ter in die. | Feb. 8, 1822—Cured. |
| 22 | Sarah Orme, 26, O.P.H.... | Ilkeston | May 8th | Bronchocele, &c. 13 y. | Pil. Cambogiæ c. gr. v. vel. x. h. s.; T. Iod. gutt. xv. ad xxx. ter in die. | Nov. 2, 1821—Cured. |
| 23 | Eliz. Dennis, 29, I.P.H. .. | Basford | May 11th | Bronchocele | Pil. Cambogiæ c. et T. Iodini gutt. xv. ter in die. | June 29, 1821—Cured. |
| 24 | Sarah Pym, 39, O.P.H..... | N. Sneinton | May 29th | Bronchocele, &c. 20 y. | Pil. Aper. et Magnes. Sulph. p. r. n.; T. Iodini gutt. xv. ad xxxv. ter in die. | Dec. 7, 1821—Cured. |
| 25 | Mrs. L——, 40, P.P....... | Arnold | June 14th | Bronchocele and Dyspnœa | Pil. Cambogiæ c.; T. Iodini gutt. xv. ter in die. | July 14, 1821—Much relieved; the medicines to be continued some time longer. |

| | | | | | | |
|---|---|---|---|---|---|---|
| 26 | Hannah Wilkinson, 39, O.P. | Gedling | June 19th | Bronchocele, &c. | Mist. Purg. p. r. n.; T. Iodini gutt. xv. ter in die. | July 13, 1821—Discharged for non-attendance. |
| 27 | Miss J——, 23 ........... | Nottingham | July 3d | Bronchocele, &c. | Pil. Cambogiæ c. gr. x. h. s.; Tæ. Iodini gutt. x. ad xl. ter in die; Linim. cum T. Iodini h. s. | October 1, 1821—Cured. |
| 28 | Catherine Joyce, 7, O.P.H. | Gotham | July 10th | L. Bronchocele & Dyspnœa, 2 y. | Magnes. Sulph. ʒij. p. r. n.; T. Iodini gutt. x. ad xv. ter in die. | Aug. 24—Much relieved; left off attending. |
| 29 | Sarah Meakin, 33, O.P.H. .. | Greasley | July 10th | Bronchocele, 16 y. M.A.L.S. | Pil. Cambogiæ c. gr. x. h. s.; T. Iodini gutt. xv. ad xl. ter in die. | Sept. 7, 1821—Bronch. nearly dissipated; left off attending—Cured. |
| 30 | Mary Goodhead, 27, P. and O.P.H. ................ | Nottingham | Sept. 19th—Pr. P. Nov. 13th | L. Bronchocele, 6 y. M. | Pil. Cambogiæ c. gr. x. h.s.; T. Iodini gutt. xv. ad xxx. ter in die. | Jan. 10, 1823—Not reduced in size; could not take the medicine in sufficient quantity. |
| 31 | Anne James, 25, O.P.H. .. | Southwell | Dec. 4th | Bronchocele, &c. 7 y. | Pulv. Rhei. c. p. r. n.; T. Iodini gutt. xx. ad xxx. ter in die. | April 6, 1822—Cured. |
| 32 | Mary Weston, 18, O.P.H. .. | Nottingham | Dec. 4th | L. Bronchocele, 4 y. | Pil. Aloes c. gr. x. h. s.; T. Iodini gutt. xx. ad xl. ter in die. | April 26—Much reduced; left off the medicines, owing to leaving Nottingham. |
| 33 | Eliz. Cummings, 13, P.P. .. | N. Radford | Dec. 31st, 1822. | Bronchocele | Pil. Aloes c. gr. x. h. s.; T. Iodini gutt. x. ad xxxv. ter in die. | April 6, 1822—Cured. |
| 34 | Sarah Scott, 17, O.P.H..... | Nottingham | Jan. 11th | Bronchocele | Pil. Cambogiæ c.; T. Iodini gutt. xx. ad xxx. ter in die. | May 17, 1822—Cured. |
| 35 | Charlotte Shaw, 8, O.P.H. | Nottingham | Feb. 15th | Bronchocele, &c. | T. Iodini gutt. vi. ter in die; Mist. Purg. p. r. n. | March 15, 1822—Cured. |
| 36 | Martha Cox, 48, O.P.H. .. | Nottingham | Feb. 12th | Bronchocele, &c. | Pil. Cambogiæ c. gr. x. h. s.; T. Iodini gutt. xx. ad xxx. ter in die. | May 31, 1822—Not reduced in size. Discharged. |
| 37 | Mrs. J——, 35, P.P. ...... | Nottingham | Feb. 28th | Bronchocele, 1 S. | Hirud. vi. tumori; Pil. Cambogiæ c.; T. Iodini gutt. xx. ter in die. | March 6, 1822—Better: cured in about 6 weeks. |
| 38 | Anne Thoresby, 18, P.P. .. | Nottingham | March 11th | Bronchocele, 5 y. M. | Magnesiæ Sulph. ʒij. m.; T. Iodini gutt. xx. ad xxx. ter in die. | Mar. 23—Tumor smaller. Left off the medicines. |
| 39 | Eliz. Booth, 31, O.P.H. .... | Nottingham (Native of Derbyshire) | March 19th | Bronchocele, 26 y. &c. | Pulv. Jalapæ c. ʒj. mane p. r. n.; T. Iodini gutt. xx. ter in die, &c. | May 17, 1823—Cured. |

| No. | Name and Age. | Residence. | Admitted, 1822. | Disease, &c. | Treatment, &c. | When and how Discharged. |
|---|---|---|---|---|---|---|
| 40 | Anne Daykin, 36, O.P.H. ·· | Lenton | April 6th | L. Bronchocele | Pil. Cambog. c. p. r. n.; T. Iodini gutt. xv. ad xl. ter in die. | August 30, 1822—Cured. |
| 41 | H. Wollatt, 17, O.P.H. ···· | Hucknall | April 9th | Bronchocele (return of) | Pil. Aloes c. gr. x. h. s.; T. Iodini gutt. xx ad xl. ter in die. | Oct. 11, 1822—Cured. |
| 42 | Mary Overin, 34, O.P.H. ·· | Nottingham (from Derbyshire) | April 30th | Bronchocele and Dysphagia | Pil. Cambog. c. gr. x. h. s.; T. Iodini gutt. xx. ad xxx. ter in die. | June 20, 1822—Cured. |
| 43 | Elizabeth Fox, 47, O.P.H. | Nottingham | April 50th | Bronchocele, 20 y. | Pil. Cambog. c. gr. x. h. s.; T. Iodini gutt. xx. ad xlv. ter in die. | April 18, 1823—Cured. |
| 44 | Miss H. T. 21, P.P. ······ | Nottingham | May 5th | Bronchocele, 13 y. Dyspnœa, &c. | Pil. Camb. c. gr. x. p. r. n.; T. Iodini gutt. xv. ad xxxv. ter in die. | June 23, 1822—Cured. |
| 45 | Fanny Kitchen, 16, P.P. ·· | Nottingham | July 4th | Bronchocele, &c. | Pil. Cambog. c. gr. v. h. s.; T. Iodini gutt. xx. ter in die. | Has not returned. |
| 46 | Hannah Pollard, 15, P.P. ·· | Nottingham | August 26th | Bronchocele | Pil. Aloes c. gr. x. h. s.; T. Iodini gutt. xx. ad xxx. ter in die. | Nov. 28, 1822—Cured. |
| 47 | Mary Smith, 48, O.P.H.····· | Lenton | Sept. 24th | Bronchocele, 8 y. &c. | Mist. Purg. ℥ ij. m. p. r. n. capt.: T. Iodini gutt. xx. ter in die. | Dec. 27, 1822—Cured. |
| 48 | Mary Tomlinson, 20, P.P. | Nottingham | October 17th | Bronchocele | Pil. Cambog. c. gr. v. ad xv. h. s.; T. Iodini gutt. xv. ter in die. | Has not returned. |
| 49 | Elizabeth Mart, 14, P.P. ·· | Nottingham | October 17th | Bronchocele, 2 y. M. S. | Pil. Cambog. c. gr. x. h. s.; T. Iodini gutt. xv. ad xx. ter in die. | Dec. 16, 1822—Cured. |
| 50 | Mr. Wm. B——, 41, P.P.·· | Over Broughton | October 18th | L. Bronchocele, 30 y. | Pulv. Purg. p. r. n.; Mist. Tonica; T. Iodini gutt. xv. ad xxx. ter in die. | April, 1823—Cured. |
| 51 | Francis Soar, 15, O.P.H. ·· | N. Radford | Nov. 5th | L. Bronchocele, Dyspnœa, &c. | Magnes. Sulph. ʒij. m. p. r. n.; T. Iodini gutt. xv. ad xxv.; et Ung. Iodini. | Sept. 5, 1823—Much reduced; will not take the medicines. Discharged. |
| 52 | Hannah Elliott, 26, P.P. ·· | N. Radford | Dec. 18th | Bronchocele, &c. | Pil. Cambog. c. gr. x. h. s.; T. Iodini gutt. xv. ad xliij. ter in die. | April 24, 1823—Cured. |
| 53 | Ellen Whiting, 18, P.P.···· | Bunny | Dec. 21st | L. Bronchocele, 9 y. | Pil. Aloes c. gr. x.; T. Iodini gutt. xv. ad xxxv. ter in die; Linim. cum T. Iodini. | Feb. 18, 1823—Cured. |
| 54 | Miss Ann P. 19, P.P.······ | Nottingham | Dec. 23d | Bronchocele, &c. | Tinct. Iodini gutt. xij. ad xx. ter in die. | Feb. 18, 1823—Cured. |

| | | | | | | |
|---|---|---|---|---|---|---|
| 55 | Miss S——, 30, P.P. …… | Nottingham | Jan. 8, 1823 | Bronchocele, &c. | Pil. Aper.; T. Iodini gutt. xv. ad xx. ter in die. | Feb. 20, 1823—Cured. |
| 56 | Sarah Gibson, 26 …… | Nottingham | Feb. 24th | L. Bronchocele, 8 y. | T. Iodini gutt. xv. ad xx. ter in die. | Feb. 27, 1823—Has not returned; result unknown. |
| 57 | Ann Osborn, 14, P.P. …… | Nottingham | Feb. 26th | Bronchocele, 3 y. and Dyspnœa | Pil. Aloes c. gr. x. h. s.; T. Iodini gutt. xv. ad xxx. ter in die. | Oct. 22, 1823—Cured. |
| 58 | Elizabeth Joynes, 15, P.P. | Nottingham | Feb. 27th | Bronchocele, 1 y. 2 p. A. | T. Iodini gutt. xv. ad xx. | March 6, 1823—Has not returned. |
| 59 | Sarah Hardy, 17, P.P. …… | Ruddington | March 3d | Bronchocele, 4 y. &c. | Magnes. Sulph. ℥ij. mane; T. Iodini gutt. xv. ad xxx. ter in die. | March 26, 1823—Much reduced in size. Has not returned since. |
| 60 | Susan Marshall, 15, P.P. …… | Nottingham | March 4th | Bronchocele, 4 y. | Pil. Camb. c. gr. x. p. r. n.; T. Iodini gutt. xv. ter in die. | March 10, 1823—Better. Has not returned. |
| 61 | Sarah Osborne, 17, O.P.H. | Hucknall | March 11th | Bronchocele, 4 y. | Pil. Camb. c. gr. xv. h. s.; T. Iodini gutt. xv. ad xxv. ter in die. | May 30—Cured. |
| 62 | Mr. T——, 22, P.P. …… | Nottingham | March 19th | L. Bronchocele, M. | Pil. Camb. c. gr. x. h. s.; T. Iodini gutt. xv. ad xxxv. ter in die.—(Taken irregularly.) | July, 1823—Cured. |
| 63 | Mrs. L——, 25, P.P. …… | Nottingham | March 27th | Bronchocele, 4 y. and Dyspnœa | Pil. Cambog. c. gr. x. h. s.; T. Iodini gutt. xv. ad xx. ter in die. | April 17, 1823—Bronchocele much reduced. Has not returned. |
| 64 | German Hodgkinson, O.P.H. | Eastwood | April 1st | Bronchocele, 9 m. and Dyspnœa | Pil. Cambog. c. gr. x. h. s.; T. Iodini gutt. xv. ad xx. ter in die. | June 24, 1823—Cured. |
| 65 | Lydia Barton, 35, O.P.H. …… | Hucknall Common | April 1st | Bronchocele, 6 y. and Dyspnœa | Pil. Cambog. c. gr. x. h. s.; T. Iodini gutt. xx. ad xxx. ter in die. | November 17—Cured. |
| 66 | Francis Wainwright, 37, O.P.H. …… | Hucknall (Native of Derbyshire) | April 1st | Bronchocele | Pil. Cambog. c. gr. xv. h. s.; T. Iodini gutt. xx. ter in die. | May 30, 1823—Cured. |
| 67 | Mary Beeston, 18, P.P. …… | Radford | April 12th | Bronchocele, 2 y. and Lepra vulg. | Pil. Cambog. c. gr. x. h. s.; T. Iodini gutt. xv. ad xx. ter in die. | June 28, 1823—Cured. |
| 68 | Mrs. S——, 53, P.P. …… | Nottingham | April 20th | Bronchocele, 5 y. | T. Iodini gutt. xv. ad xx. ter in die, et Magnes. Sulph. ℥ij. p. r. n. | July 23—Cured. |
| 69 | Miss Sarah S——, 15, P.P. | Nottingham | April 20th | Bronchocele, 1½ y. | T. Iodini gutt. xv. ad xx. ter in die; Pil. Camb. c. gr. x. h. s. p. r. n. | October 4—Cured. |
| 70 | Miss Jane S——, 18, P.P. …… | Nottingham | April 20th | Bronchocele, 6 m. | T. Iodini gutt. xv. ter in die; Pil. Cambog. c. gr. x. p. r. n. | May 28—Cured. |

| No. | Names and Age. | Residence. | Admitted, 1823. | Disease, &c. | Treatment, &c. | When and how Discharged. |
|---|---|---|---|---|---|---|
| 71 | Sarah Davis, 39, O.P.H. .... | Hucknall | April 22d | Bronchocele, &c. | Pil. Hydr. gr. x. m. et h. s.; T. Iodini gutt. xv. ter in die. | May 23, 1823—Bronchocele almost dissipated. Cured. |
| 72 | Ann Sharp, 18, P.P. ...... | Worthington (Leicestershire) | May 6th | Bronchocele, S. | T. Iodini gutt. xv. ad xx. ter in die; Magn. Sulph. ʒ ij. alter matut. | Sept. 2, 1823—Cured. |
| 73 | Miss C——, 32, P.P. ...... | Nottingham | May 8th | Bronchocele, 15 y. | Pil. Cambog. c. gr. x. h. s.; T. Iodini gutt. xv. ter in die. | Nov. 1, 1823—Cured. |
| 74 | Miss C. C——, 24, P.P. .... | Nottingham | May 8th | Bronchocele, 4 y. | Pil. Cambog. c. gr. x. p. r. n.; T. Iodini gutt. xv. ter in die. | Sept. 15, 1823—Cured. |
| 75 | Elizabeth Butler, 8, P.P. .. | Bloomsgrove | May 29th | Bronchocele, 6 m. and upwards | Pulv. Purg. p. r. n.; T. Iodini gutt. viij. ad x. ter in die. | Sept. 3, 1823—Cured. |
| 76 | Sophia Saunders, 18, P.P. | Nottingham | May 29th | Bronchocele, 5 y. F. Bronch. (from Derbyshire) | T. Iodini gutt. xij. ad xxv. ter in die; Ung. Iodini m. et h. s.; Pil. Camb. c. gr. x. h. s. p. r. n. | September 30—Cured. |
| 77 | Miss F——, 20, P.P. ...... | Nottingham | May 31st | Bronchocele | T. Iodini gutt. x. ad xv. ter in die.—(Taken irregularly.) | September 3—Cured. |
| 78 | Miss L. F——, 18, P.P. .... | Nottingham | May 31st | Bronchocele | T. Iodini gutt. x. ad xv. ter in die.—(Taken irregularly.) | Dec. 17, 1823—Cured. |
| 79 | Elizabeth Reddish, 24, P.P. | Lowdham | June 2d | L. Bronchocele, 10 y.; M. large Bronchocele | T. Iodini gutt. xv. ad xviij.—℞. Lin. Super. c. ℥ ij.; T. Iodini ʒij. M. fiat Linim. quo fricetur tumor. | July 27, 1823—Tumor much smaller. Medicines to be continued. |
| 80 | Mrs. M——, 36, P.P. .... | Nottingham | June 13th | Bronchocele, 28 y. D. | Pil. Cambog. c. gr. x. h. s.; T. Iodini gutt. xv. ad xx. ter in die. | Bronch. greatly reduced; still under treatment. |
| 81 | Maria Barnett, 17, O.P.H. | Carlton Hill | June 24th | Bronchocele, 5 y. | T. Iodini gutt. xij. ad xv. ter in die. | Oct. 5, 1823—Bronchocele much reduced. |
| 82 | Eliz. Brown, 17, O.P.H. .... | Nottingham | June 24th | Bronchocele, from a child | T. Iodini gutt. xv. ad xx. ter in die; Ung. Iodini h. s.; et Pil. Aloes c. gr. x. h. s. p. r. n. | April 2, 1824—Cured. |
| 83 | William Cale, 14½, O.P.H. . | Hucknall | August 5th | Bronchocele, 2 y. | T. Iodini gutt. xij. ad xv. ter in die; Ung. Iodini m. et h. s. tumori; Magn. Sulph. ʒij. m. p. r. n. | Nov. 7, 1823—Cured. |

| | | | | | | |
|---|---|---|---|---|---|---|
| 84 | Eliz. Selby, 14, I.P.H. .... | Lenton | August 5th | Bronchocele | Capiat T. Iodini gutt. x. ter in die; Pil. Cambog. c. gr. x. h. s. | Sept. 9, 1823—Cured. |
| 85 | Mary Anne Allen, 16, P. P. | Brewhouse Yard | August 28th | Bronchocele, 7 y. | T. Iodini gutt. xv. ad xx.; Linim. Iodini h. s. | Feb. 17, 1824—Cured. |
| 86 | Sarah Redgate, 21, P.P. .. | Leen Side | Sept. 4th | Bronchocele, 4 y. | T. Iodini gutt. xv. ter in die; Magn. Sulph. ℥ss. p. r. n. | Has not returned. |
| 87 | Elizabeth Peet, 14, P.P. .. | Radcliffe | Sept. 27th | Bronchocele, 1 y. | T. Iodini gutt. xij. ter in die ex aqua. | Has not returned. |
| 88 | Sarah Pickering, 16, P.P... | N. Radford | October 6th | L. Bronchocele, 4 y. | T. Iodini gutt. xv. ad xx.; Hirud.; Magnes. Sulph. ʒij. p. r. n. | March 10, 1824—Bronchocele much smaller; still under treatment. |
| 89 | Charles Birkett, 7, O.P.H... | Linby | October 7th | Bronchocele, 6 m. | Magnes. Sulph. ʒj. mane p. r. n.; T. Iodini gutt. vij. ad viij. ter in die. | Feb. 6, 1824—Cured. |
| 90 | Sarah Smith, 15, P.P. .... | Bunny | October 16th | Bronchocele, 4 y. 2 S. M. 2 p. A. | T. Iodini gutt. xv. ad xxv.; Magnes. Sulph. p. r. n. | Jan. 10, 1824—Cured. |
| 91 | Harriet Taylor, 12, P.P. .. | Brinsley | October 22d | Bronchocele | T. Iodini gutt. vj. ad viij.; Magnes. Sulph. ʒj. p. r. n. | Jan. 11, 1824—Cured. |
| 92 | Mary Bostock, 18, O.P.H. | Nottingham | October 28th | Bronchocele, 8 y. S. | Pil. Camb. c. gr. x. h. s.; T. Iodini gutt. xv. ter in die. | March 5, 1824—Cured. |
| 93 | Benj. Shipley, 55, O.P.H... | Nottingham | October 28th | L. Bronchocele, since a child | Pil. Cambog. c. gr. x. h. s.; T. Iodini gutt xv. ad xx. ter in die. | Feb. 20, 1824—Cured. |
| 94 | Ann Oats, 27, P.P. ...... | Cromford (Derbyshire) | October 30th | Bronchocele | Pil. Cambog. c. gr. x. h. s.; Magnes. Sulph. p. r. n.; T. Iodini gutt. xv. ad xviij. ter in die. | Has not returned. |
| 95 | Ann Smith, 19, O.P.H. .... | Lenton | Nov. 18th | Bronchocele (return of), and Lepra vulg. | T. Iodini gutt. xv. ter in die; Pil. Cambog. c.; et Magnes. Sulph. ʒij. m. p. r. n. | Jun. 2, 1824—Cured. |
| 96 | Francis Maltby, 60, P.P. .. | Bingham | Dec. 3d | Bronchocele, D. | T. Iodini gutt. xv. ad xx.; Pil. Camb. c. gr. x. h. s. | Has not returned. |
| 97 | Mary Richardson, 27, P.P. | Not noted down | Dec. 6th | Bronchocele, 4 y. | T. Iodini gutt. xv. ad xx. ter in die. Pil. Aper. | Has not returned. |
| 98 | Hannah Bromhead, 17, O.P.H. ................ | Nottingham | Dec. 9th | L. Bronchocele, 3 y. | T. Iodini gutt. xv. ad xx.; Pil. Aper. Linim. Iodini h. s.; Hirud. viij. bis tumori. | Bronch. much reduced. Still under treatment. |
| 99 | Ann Thoresby, 20, O.P.H. | Nottingham | Dec. 9th | L. Bronchocele, 4 y. M. | Liq. Iodini gutt. viij. ad xxiv. ter in die; Linim. Iodini; Pil. Aper. | Bronch. much reduced. Still under treatment. |

| No. | *Name and Age.* | *Residence.* | *Admitted, 1823.* | *Disease, &c.* | *Treatment, &c.* | *When and how Discharged.* |
|---|---|---|---|---|---|---|
| 100 | George Bull, 19, P.P. .... | Nottingham | Dec. 15th | L. Bronchocele, M. and S. | T. Iodini gutt. xv. ad xx.; Pil. Aper. p. r. n. | Bronch. much smaller.—Left off; but has resumed the use of the medicines. |
| 101 | Eliz. Smith, 3 and 9 months | N. Radford | Dec. 21st. | Bronchocele, 2 m. standing | Liq. Iodini gutt. iv. ad v. ter in die. Magn. Sulph. coch. j. min. p. r. n. | March 7, 1824—Cured. |
| 102 | Miss G——, 18, P. P. ...... | Nottingham | Jan. 3, 1824 | Bronchocele | Liq. Iodini gutt. viij. ad x. ter in die. Pil. Aper. p. r. n. | March 23, 1824—Cured. |
| 103 | Eliz. Warren, 32, P.P. .... | Ilkeston (Derbyshire) | Jan. 7th | Bronchocele, 23 y. | T. Iodini gutt. xij. ad xviij. ter in die; Pil. Aper. p. r. n. | Tumor much smaller;—under treatment. |
| 104 | Ann Hickinbottom, 51, O.P.H. ........ ....... | Wilford | Jan. 20th | Bronchocele, large and hard, 18 y. | Liq. Iodini gutt. x. ad xxvj. ter in die; Linim. Iodini h. s.; Pil. Aper. | Bronch. much reduced. Still under treatment. |
| 105 | J. Mellows, 4, O.P.H. .... | Linby | Jan. 20th | Bronchocele, &c. 5 m. | Liq. Iodini gutt. iv. ad vj. ter in die; Magnes. Sulph. coch. j. minimum p. r. n. | April 13, 1824—Cured. |
| 106 | Ann Watson, 34, P.P. .... | Nottingham (from Derbyshire) | Jan. 27th | Bronchocele since a child | T. Iodini gutt. xij. ad xx. ter in die; Lin. Iodini h. s. | Bronch. not much smaller. Still under treatment. |
| 107 | Mary Flinders, 16, O.P.H. | Arnold | Feb. 10th | Bronchocele, 4 y. F. M. and p. A. | Liq. Iodini gutt. x. ad xviij. ter in die. | Bron. smaller and softer. Still under treatment. |
| 108 | Jane Everett, 21, I.P.H. .. | Nottingham (born in Rutlandshire) | Feb. 10th | Bronchocele, 11 y. | Liq. Iodini gutt. x. ad xv. ter in die; Mist. Purg. p. r. n. | April 13, 1824—Cured. |
| 109 | Mary Humphries, 45, P.P. | Lenton | Feb. 28th | Bronchocele, several y. standing | T. Iodini gutt. xij. ter in die; Pil. Aper. p. r. n. | Under treatment. |
| 110 | Isaac Knowles, 24, O.P.H. | Heanor (Derbyshire) | March 2d | L. Bronchocele, 9 y. | Liq. Iodini gutt. xv. ter in die; Mist. Purg. p. r. n.; Linim. Iodini. | Bronch. much smaller.—Still under treatment. |
| 111 | A. Knutteries, 15, I.P.H. .. | Southwell | March 6th | Bronchocele, 4 y. | Pulv. Rhei. c. ʒss. p. r. n.; Liq. Iodini gutt. viij. ter in die. | Discharged at her request. March 27, 1824. |
| 112 | Mary Goodhead, 30 ...... | Nottingham | March 8th | L. Bronchocele, 9 y. M. | Solut. Iodini gutt. xv. ad xx. ter in die; Lin. Iodini h. s. | Bronchocele softer. Under treatment. |
| 113 | William Leavers, 9, P.P. .. | Greasley | March 10th | L. Bronchocele, 15 m. | Liq. Iodini gutt. xiij. ter in die; Magnes. Sulph. ʒj. p. r. n. | April 20, 1824—Under treatment. |

| | | | | | | |
|---|---|---|---|---|---|---|
| 114 | Mary Herbert, 13, P.P. .... | N. Radford | March 10th | Bronchocele, &c. M. | Liq. Iodini gutt. x. ter in die; Magn. Sulph. ℨij. p. r. n. | Under treatment. |
| 115 | Maria Bamford, 18, P.P. .. | Nottingham | March 15th | Bronchocele, 4 y. | Liq. Iodini gutt. x. ter in die; Mag. Sulph. ℨij. p. r. n. | Under treatment. |
| 116 | Martha Rice, 16, P.P. .... | Ilkestone (Derbyshire) | March 20th | Bronchocele, 4 y. M. B. S. | Liq. Iodini gutt. xij. ter in die, ex aqua. | May 1, 1824—Cured. |
| 117 | Rebecca Lovett, 15, P.P. .. | Nottingham | March 20th | Bronchocele, 2 y. | Liq Iodini gutt. x. ter in die; Magn. Sulph. ℨij. mane p. r. n. | April 24, 1824—Cured. |
| 118 | Susan Maltby, 23, P.P. .... | Nottingham | March 22d | Bronchocele, right side of Thyroid Gland, &c. | Pil. Cambog. c. gr. x. h. s.; Liq. Iodini gutt. viij. ad x. ter in die. | Under treatment. |
| 119 | Mary Carrington, 19, lace runner, O.P.H. ........ | Nottingham | March 23d | Bronchocele, 5 y. | Pil. Camb. c. gr. x. p. r. n.; Liq Iodini gutt. viij. ter in die. | May 13, 1824—B. much smaller and softer. Still under treatment. |
| 120 | Ann Hall, 14, servant, O.P.H. ...... ......... | Nottingham | March 23d | Bronchocele, 1 y. —Catamenia regular. | Liq. Iodini gutt. viij. ad xviij. ter in die. Pil. Cambogiæ c. gr. x. h. s. p. r. n. | April 23, 1824—B. softer. Under treatment. |

### GENERAL ABSTRACT.

Total number of Cases .................................................. 120
From these deduct four Females twice admitted ......................... 4

Number of individual Cases .................. 116

Viz.—MALES, cured ............................ 10

———, much relieved ........................ 1

———, discharged for non-attendance ........ 1

———, improving under treatment ............ 3

} Total, 15

FEMALES, cured ........................... 66

————-, much relieved ................... 9

————-, without relief .................. 2

————-, discharged for non-attendance ...... 10

————-, improving under treatment ......... 14

} Total, 101

Number of individual Cases as stated above ............ 116

### *Remarks on some of the preceding Cases.*

(The number of the Remark corresponds with the number of the Case.)

No. 18.—This case will be given in detail, under the head of Scrofula. It was the first instance of the beneficial effects of iodine in reducing enlarged lymphatic glands, that had come under my notice. Respecting the goitre in this instance, nothing deserving particular notice occurred ; but it may not be uninteresting to those who are in the habit of employing the tincture of iodine as a medicine, to know that this young woman swallowed, by mistake, nearly an ounce of the tincture, of the strength employed by myself, without being diluted : it was immediately rejected by vomiting; and I am happy to add, that no inconvenience or bad effects ensued.

No. 27.—This is the first case in which I employed iodine externally. To one ounce of the liniment. saponis comp. of the London Pharmacopœia, I add one drachm of the tincture of iodine to form a liniment which most patients can bear to be rubbed into the tumor once a-day, and some morning and evening; but in some persons the skin is apt to become tender, and disquammation of the cuticle to take place, from using the liniment twice a-day: the frequency of its application must, therefore, be regulated according to what the skin will bear in different individuals. This is a very convenient form of exhibiting the iodine externally; and it has the advantage of allowing the preparation to be kept stopped up in a phial, thereby preventing the evaporation of the iodine, which takes place when the tincture of iodine is mixed with hog's lard, to form an ointment. I have not yet had occasion to employ the hydriodate of potass in the form of ointment, and cannot, therefore, speak as to its efficacy from personal observation ; but I mean to avail myself of those opportunities that daily occur to me, to try its comparative powers, and to ascertain, as far as I am able, the advantages and inconveniences, if any, attending its exhibition.

I had almost omitted to remark, that the tincture of iodine blanches the compound soap liniment with which it is mixed. Is this owing to the iodine attracting hydrogen, and disengaging oxygen from some substance of which these gases are constituent parts? or is it owing to some inherent property of the iodine itself? The professed chemists must settle this point.

No. 28.—This was a very large bronchocele in so young a patient, and, for six months before applying for medical assistance, the goitre, by pressing on the trachea, occasioned great difficulty of breathing, with wheezing and hoarseness. In ten days the tumor was much softer, and measured half an inch less, and the distressing symptoms were very much alleviated. After finding herself at ease, she left off attending at the hospital, and I saw nothing more of her till the beginning of April last, when a heavy snow-storm forced me to seek shelter in a cottage near the village where she resides. Here I found her, and was happy to see that the goitre was nearly dissipated; and I learnt from her that it continued gradually to diminish in size for a long time after she had left off the tincture of iodine. At the time I saw her, she experienced no inconvenience from the remains of the goitre.

No. 29.—No impression could be made on the bronchocele in this case, till the patient took forty drops of the tincture of iodine three times a-day. The tumor was nearly dissipated in the course of two months. In this case, the tincture of iodine occasioned a good deal of disorder of the system, with loss of flesh and strength, owing to the dose of the medicine having been too large for her. Greater experience has since taught me that a cure, in all probability, might have been accomplished in this case by the exhibition of smaller doses of the medicine, if continued for a sufficient length of time. I thought it necessary to leave off the iodine, at least for a time. The patient did not return to the hospital; but I learnt from a neighbour of her's, some time after, that she was well, and cured of the goitre.

No. 30.—This woman first applied to me as a gratuitous patient; but, owing to the expense of the medicine, she got recommended as an out-patient of the General Hospital, and came under my care. For a short time she took thirty drops of the tincture of iodine three times a-day; but it occasioned so much sickness and disorder of the stomach, that I was obliged to reduce the dose to fifteen drops three times a-day; and even in this small dose the stomach was affected with more or less nausea and disorder, and no sensible effect was produced on the goitre. In this case, too, the menstrual discharge, that had always been very regular and rather copious, disappeared for some time; owing, I believe, to the debility and disorder of the system, induced by the iodine in this patient, from idiosyncrasy of constitution. I may observe, that her mother laboured under a large goitre, of sixteen or seventeen years' standing, that had resisted the use of burnt sponge. She recommended her mother to use the same drops that I had prescribed for herself, and she took them, and was cured of the goitre; at the same time her general health was improved in a very marked degree. This information I received from my patient, who has often expressed her surprise that the same medicine should have such opposite effects on her mother and herself, when the dose was the same.

This woman applied to me again on the 8th of March, 1824, to endeavour to dissipate the goitre, as it had increased in size, and also in hardness, since she left off the use of the iodine; which, it appears, kept the tumor from enlarging, if it could not accomplish a cure, owing to the small quantity she was able to take. I prescribed fifteen drops of the solutio iodini, to be taken in water three times a-day; and, for the last week, she has taken twenty drops of the solution of iodine three times a-day, without inconvenience, and for the same period has rubbed the goitre with linimentum iodini, every night at bed-time. The patient, her husband, and myself, agree in opinion that the goitre is softer, but it was not sensibly smaller when measured. As the patient is anxious to be cured, I mean to give the hydriodate of potass a fair trial in this case, both internally and externally, before I give up the case as incurable.

No. 41.—This patient experienced a return of bronchocele, eight months after being cured by the internal use of iodine, as detailed in the first case. She has again been cured by the same means; and I hope that the cure will now be more permanent, from the circumstance that the menses appeared, for the first time, a few days before the goitre was last dissipated. The water she was in the habit of using is very hard; I therefore advised her only to take rain-water internally.

No. 44.—This young woman breathed with difficulty, and wheezed very much, when she ascended a stair or walked quick. She experienced great relief at the end of fourteen days, and felt quite well in thirty-seven days; but the tincture of iodine was given twelve days longer, to prevent a relapse. The only inconvenience that this patient complained of, was that the tincture of iodine burnt her throat; but she was quite delighted at the rapidity of the cure, as she fully believed she was going into a consumption when she first applied to me.

No. 46.—The thyroid gland began to enlarge when the patient was three years of age. Three paternal aunts labour under the same disease. The goitre was dissipated without the catamenia having ever appeared. Her mother was eighteen before she menstruated, and perhaps the daughter may take after her in that respect. The drops occasioned a little sickness at stomach after taking them, but it soon went off, and the patient found no other inconvenience.

No. 49.—This young woman laboured under goitre, and came under my care, as an out-patient of the hospital, the 6th of June, 1820, and was discharged cured on the 8th of September following, by taking the following medicines:—Pulv. Jalapæ c. ℈ij. omni mane, p. r. n.

℞. Spongiæ Ustæ.

Syr. Zinzib. āā ℥j. M. ft. Elect. cujus capiat coch. j. minimum (tea-spoonful) ter in die.

The goitre has since returned, and was firm to the touch, and large than a goose's egg, and, by pressing on the trachea, occasioned short ness of breath and wheezing. When re-admitted, she was cured in two months, by the internal use of the tincture of iodine. Catamenia regular, and not affected by the medicines exhibited.

No. 62.—This young man is of a delicate constitution, and laboured under a large bronchocele, which is now nearly dissipated. There is nothing unusual to remark in this case, except that soreness of the gums, and a very considerable ptyalism, came on during the exhibition of the iodine, and that it was necessary to restrain the discharge by the use of an alum gargle.

No. 64.—This patient laboured under dyspnœa, attended with a great degree of wheezing, which first attracted the attention of his parents, and led them to examine his neck. The bronchocele was about the size of a hen's egg, and, by pressing on the trachea, occasioned the distress in breathing. In the course of four days the breathing was relieved; and in thirty-two days the tumor was nearly dissipated. The tincture of iodine was continued some time longer, to complete the cure and prevent a relapse.

Nos. 68, 69, and 70, refer to a mother and two daughters. I have nothing particular to remark, except that the mother, a native of Rutlandshire, remained free from the disease till five years previous to the time she applied to me, although she has resided twenty-six years in this town. The daughters seem to have had a stronger disposition to the disease than the mother. Their general health was improved, and they suffered no inconvenience whatever from the iodine.

No. 71.—I find that the tincture of iodine, given in doses of fifteen drops, occasioned so much giddiness in this case, that the patient was at first obliged to keep in bed for two or three days; but, after it had been taken for a week, that it agreed very well.

No. 72.—I have nothing particular to remark respecting this case; but it may be worth while to observe, that a sister of her's, who laboured under bronchocele, got rid of it, when travelling in Ireland and Scotland, without using any means to dissipate the tumor. This information I received from my patient.

Nos. 73 and 74 refer to the cases of two sisters. They took the iodine very irregularly, and at considerable intervals of time, otherwise they might have been sooner cured. The mother has the left side of the thyroid gland very much enlarged, but, being upwards of sixty-five years of age, she does not wish to try any means to dissipate it.

Nos. 77 and 78—I am disposed to notice, being the cases of two sisters of delicate constitutions, in which the tonic effects of the iodine was so remarkable, that it did not escape the observation of the mother of the young ladies, who is a woman of acute discernment and sound understanding, and, being an invalid, she consulted me as to the propriety of her taking some of the same medi[illegible]e herself, with a view to its tonic effects on her own system. To obtain tonic effects from iodine, it is necessary to exhibit it in small doses, so as not to overpower and disorder the system, and to preserve a regular and open state of the bowels.

No. 80.—This is a case of long standing, but it is gradually yielding to the use of the iodine, which is now taken in doses of fifteen drops two or three times a-day, occasionally omitting it for a week. This patient has often remarked to me, how much her general health has been improved since she began to take the iodine.

No. 81.—This young woman began with twelve drops of the tincture of iodine in water, and after ten days the dose was increased to fourteen drops. After continuing the medicines for a month, it is stated that the goitre is much smaller and softer, and measures about an inch less; and that the tincture of iodine occasioned some dizziness and head-ache. Her bowels tardy, having only one stool in twenty-four hours: ordered to take three aperient pills.

The next report mentions that the sickness, after taking the drops, only continued about ten minutes. Bowels continued tardy, and two drachms of the sulphate of magnesia were ordered to be taken every second morning, in addition to the pills; being convinced that the head-ache and dizziness depended, in a great measure, on the tardy state of the bowels. The last time the patient made her appearance at the hospital, she told me that she had not taken any of the tincture of iodine for fourteen days, owing to its making her dizzy, and occasioning pain and heaviness in the head; and she stated that she was so ill in the morning, with lowness and faintness, that she was unable to follow her employment of lace-mender. Under these circumstances, I ordered the aperient medicines to be continued, and directed five drops of the liquor iodini to be taken three times a-day, instead of the tincture.

The patient did not again return to the hospital, and therefore I

know nothing more of the case; but I thought it necessary to point out this case, as one in which the tincture of iodine occasioned unpleasant symptoms in a very small dose, the largest quantity ordered being fourteen drops (equal to seven drops of Dr. Coindet's tincture,) three times a-day. I cannot divest myself of the idea that the head-ache and dizziness in this case were owing, in a considerable degree, to the tardy state of the patient's bowels, notwithstanding the use of aperient medicines; but I also am disposed to believe that there is some peculiarity in this patient's constitution, that precludes the possibility of administering the tincture of iodine to the same extent that, from the table, it will appear was done in other cases, under apparently the same circumstances, not only without inconvenience, but with evident and positive advantage. Even in the small doses exhibited in this case, the goitre had subsided very considerably, and had become soft (always a favourable symptom), when I last saw this patient; and I am of opinion that, if she had continued her attendance, the cure might have been completed by frictions and small doses of the mildest preparations of iodine, without her suffering any material inconvenience.

No. 83.—This youth began with fifteen drops of the tincture of iodine three times a-day, and in twenty-four days he wheezed very little, although he did so loudly when he first came under my care. About this time the tincture of iodine made him sick, and occasioned some degree of giddiness, which was not the case when he first began to use it. He also employed the following ointment:—℞. Tinct. Iodini ʒjss.; Adipis Præp. ℥j. M. ft. Unguentum quo fricetur tumor m. et h. s.

In some individuals, after the preparations of iodine have been given internally for some time, they are apt to occasion head-ache, giddiness, sickness at stomach, with some degree of nausea, languor, and inaptitude for exertion. When these unpleasant sensations and effects occur, the best plan to remove or obviate them is to suspend for a time the use of the medicine, or to reduce the dose, as may seem most expedient. A reduction of dose, from fifteen to twelve drops, was the plan adopted by me on this occasion. The patient afterwards went on to the completion of the cure, without any uncomfortable sensations. An enlarged conglobate gland in the neck subsided very considerably during the exhibition of the iodine, but not so rapidly as the enlarged thyroid gland. His general health was greatly improved, and he admitted that he was perfectly well at the time of his discharge. On the 20th of January, 1824, he came to the General Hospital to return thanks, and then remained free from complaint.

No. 99.—This young woman's name will be found at No. 38. She took the tincture of iodine for a short time with benefit, but, owing to the expense of the medicine at that time (thirty-six shillings the ounce being the wholesale price,) she gave it up, and afterwards became an out-patient of the General Hospital, as will be seen by inspecting the table. The bronchocele is gradually subsiding and becoming softer, and there is no doubt that a cure will be accomplished in time, provided she will persevere in the use of the iodine. As showing the influence of some local cause in the production of goitre, I may mention that this young woman's mother was born and brought up in Rutlandshire, where she and all her relations were free from bronchocele; but since her marriage she has resided constantly in this town, and now labours under a very large goitre.

As I should be considered tedious by offering more remarks on individual cases of bronchocele, I may now generally observe, that in the other cases, so far as I had an opportunity afforded me of following up the treatment by the internal exhibition of iodine, they all went on favourably, without any particular unpleasant effect from the medicine, and without any injury to the constitution. On the contrary, the tincture of iodine, and other preparations of it, employed by myself, had generally a cordial and tonic effect, unless given in too great a dose; and the patients to whom it was exhibited, with the exception of the persons mentioned in the remarks, generally found themselves in better health and spirits than they had previously been for years. This observation applies not only to those who labour under bronchocele, but also (as will appear from the sequel) to those who laboured under other diseases, in which iodine was exhibited.

It is a great satisfaction for me to be able to state, that the patient (No. 30), with whom the iodine disagreed the most, and who derived the least benefit from it, has again put herself under my care (see No. 112), and is taking small doses of the hydriodate of potass, and using the linimentum iodini externally, not only without inconvenience, but with some advantage. I think it necessary to point to the above circumstance in a particular manner, as it appears, from the report of a celebrated author and reviewer, that Dr. Coindet now considers the internal exhibition of iodine as injurious, and uses it only externally, in the way of friction. I apprehend that Dr. Coindet has exhibited this powerful medicine in too large doses for the generality of patients; otherwise he could have been under no alarm about its internal use.

The rule to be observed with iodine, as with all other powerful medicines, is to begin with a small dose at first, and to increase it cautiously and at proper intervals. In this way we shall avoid doing harm, and shall be able to discover any peculiarity of constitution before the dose of the medicine can amount to a quantity sufficient to injure the constitution, or even to occasion much inconvenience.

The cases and the table will show the doses of the different preparations of iodine employed by me, as well as the ages of the patients, &c. I beg particularly to observe, that it is absolutely necessary to keep the bowels in an open and regular state during the exhibition of iodine.

The power of iodine over the absorbent system has been noticed by Dr. Coindet and others: it early attracted my attention, as will be seen by referring to the remarks No. 18. Since that time, as will afterwards appear, I have had ample proof of its great power over the lymphatic system; but I am of opinion that it exerts no peculiar or specific power over that system of vessels and glands, but that they only participate in its general energetic effects on the whole body.

I cannot suppose that a temporary excitement given to the absorbents would remove large bronchoceles, and prevent their return, unless the state of the system that gives rise to goitre was corrected. When that is accomplished, the absorbents perform their office with effect, and the swelling disappears; but, in my opinion, it is incorrect to ascribe the whole cure to increased absorption.

Before concluding my remarks, and bringing to a conclusion what I have to communicate on the subject of iodine, I can, without hesitation, state that I have found it a much more convenient and effectual remedy in dissipating goiterous swellings than burnt sponge, which I had employed, very frequently with success, for nine years before I knew any thing of the remedial powers of iodine. Independent of burnt sponge being a bulky and unpleasant article to take, it is very clear that we never can know the quantity of iodine contained in any given quantity of this medicine, or even whether it contains any iodine or not. Burnt sponge must, therefore, be considered as a very uncertain remedy, compared with iodine, and will, I have no doubt, soon give place to it.

In confirmation of the beneficial effects of iodine in the cure of bronchocele, I may state that Mr. Jowett, a highly intelligent young surgeon, who has for some time performed the duty of resident medical surgeon at St. Mary's Hospital and Dispensary, of this town, lately informed me that he has cured two cases of bronchocele by the internal exhibition of the tincture of iodine, without inconvenience to the patients, or any injury to the general health.

Before finishing what I have at present to communicate on the effects of iodine in bronchocele, I may remark that, since the above observations were sent to the press, a great many patients, from Derbyshire and other parts of the country, have applied to me for the cure of bronchocele; and I find iodine not only to support its character in the cure of goitre, but even to exceed what could reasonably have been expected from it as a remedial agent, as the following statement will fully show:—

Fanny Booth, of Cotmanhay, in Derbyshire, applied to me the 14th of April, 1824, afflicted with a very large bronchocele. I prescribed ten drops of tincture of iodine, to be taken in water three times a-day, and directed the tumor to be well rubbed every night with the iodine liniment. In fourteen days the neck and goitre measured nearly two inches less than when she applied to me.

I think it necessary to make this brief notice of Booth's case, because, in consequence of the benefit that she experienced in so short a time from the means employed, Millicent Richards, a friend and acquaintance of hers, who laboured under an enormous bronchocele, presented herself to me for cure. I give the case as it is taken down in my Journal:—

Nottingham; 1st May, 1824.—Millicent Richards, æt. sixty-seven; married and has a family; was born and brought up at Ilkeston, in Derbyshire, and has always resided there. For thirty-five years has laboured under bronchocele: at first it increased very slowly, but for the last ten or fifteen years it has increased very much, and is now of an enormous size and globular form, slightly flattened. The surface is smooth and uniform, skin tense, and of nearly the same colour as the adjoining skin. The goitre has escaped from its natural situation in the neck, and the most depending part of it now reaches as low as the ensiform cartilage, and the upper part of it is on a level with the upper part of the sternum; it is attached to the neck by a stem about three inches long, which measures twenty-two inches in circumference. The greatest circumference of the goitre is longitudinally as to the body, and measures exactly twenty-nine inches; and the shortest circumference, taken at right angles to the former, or in the transverse direction of the body, measures exactly twenty-seven inches and three-quarters, so that the tumor is nearly globular, or within one inch and a quarter of being so, and rests on the breast, suspended to the neck by the stem. To give some idea of the size of the tumor, compared with the head, I measured the latter, in a line horizontal to the upper part of the orbits, and found the head to measure exactly twenty-two inches and a half in circumference. Her general health is pretty good.

The poor woman is anxious to have the tumor reduced, and, being willing to try what iodine can accomplish in this case, I have prescribed the following medicines:—

Capiat Pil. Cambogiæ c. gr. x. omni nocte h. s.

℞. Tæ. Iodini ℥ss. Capiat gutt. xij. ter in die ex aqua.

Sumat Magnesiæ Sulphatis, ʒij. mane p. r. n.

℞. Linim. Sapon. c. ℥ij.; Tincturæ Iodini ʒij. Misce, fiat Linimentum quo fricetur tumor omni nocte hora somni.

May 22.—Has employed the medicines exactly as directed. The patient observes that the swelling must be smaller, as the skin is now quite loose, and she takes it up in folds. On re-measuring the bronchocele, I find the longest circumference rather less than twenty-eight inches, and the shortest twenty-six inches and five-eighths; so that the tumor has diminished an inch or upwards, in all directions, in the course of three weeks. Medicines agree very well. Bowels are costive; makes urine with difficulty, owing, I believe, to the constipated state of the body. The aperient pills do not move her bowels, and she has neglected to take the Epsom salts.

℞. Contr. Pilul. Cambogiæ c. Sumat Magnes. Sulphatis ʒij. vel iij. mane p. r. n. Capiat Tæ. Iodini gutt. xv. ter in die.

Fricetur tumor linimento iodini omni nocte hora somni.

I have directed her to make a bag to lace upon the tumor, so as gently to compress it, supporting the compressing bag and tumor by a kind of waistcoat. If my directions are properly carried into effect, the pressure on the tumor will, I have no doubt, assist in promoting its absorption.

Highly as I estimate the powers of iodine in the cure of bronchocele and of other diseases, I must acknowledge that, in this prodigious enlargement of the thyroid gland, its remedial powers have far exceeded what I could reasonably expect, in the short time it has been employed.

